# 4-(1,3-Benzothia­zol-2-yl)-5-methyl-2-phenyl-1-propynyl-1*H*-pyrazol-3(2*H*)-one

**DOI:** 10.1107/S1600536810040328

**Published:** 2010-10-20

**Authors:** Imane Chakib, Abdelfettah Zerzouf, Hafid Zouihri, El Mokhtar Essassi, Seik Weng Ng

**Affiliations:** a Laboratoire de Chimie Organique Hétérocyclique, Pôle de Compétences Pharmacochimie, Université Mohammed V-Agdal, BP 1014 Avenue Ibn Batout, Rabat, Morocco; b Department of Chemistry, University of Malaya, 50603 Kuala Lumpur, Malaysia

## Abstract

The title compound, C_20_H_15_N_3_OS, is a 1*H*-pyrazol-3(2*H*)-one having aromatic 4-(1,3-benzothia­zol-2-yl) and 2-phenyl substit­uents. The five-membered ring and the fused-ring system are close to planar, the r.m.s. deviations being 0.025 and 0.005 Å, respectively. The five-membered ring is aligned at 67.5 (1)° with respect to the phenyl ring and at 4.7 (1)° with respect to the fused-ring system. In the crystal, adjacent mol­ecules are linked through the acetyl­enic H atom by a C—H⋯O hydrogen bond into *C*(8) chains propagating in [010].

## Related literature

For the structure of a similar compound, 4-(benzo[*d*]thia­zol-2-yl)-2-allyl-3-methyl-1-phenyl-1,2-dihydro­pyrazol-5-one, see: Chakibe *et al.* (2010 ▶). For the structure of a related compound, (*E*)-4-(2,3-dihydro-1,3-benzothia­zol-2-yl­idene)-3-methyl-1-phenyl-1*H*-pyrazol-5(4*H*)-one, see: Chakib *et al.* (2010 ▶).
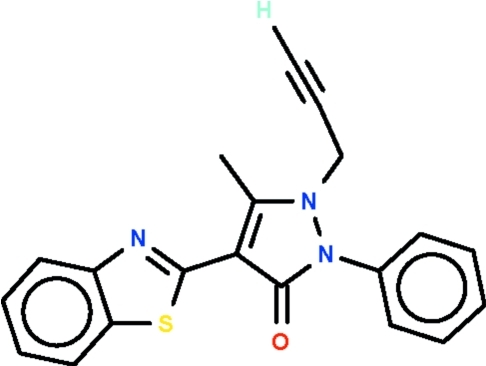


## Experimental

### 

#### Crystal data

C_20_H_15_N_3_OS
*M* *_r_* = 345.41
Orthorhombic, 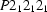

*a* = 4.8221 (1) Å
*b* = 9.3698 (2) Å
*c* = 37.6990 (9) Å
*V* = 1703.32 (6) Å^3^
*Z* = 4
Mo *K*α radiation
μ = 0.20 mm^−1^
*T* = 293 K
0.40 × 0.20 × 0.20 mm

#### Data collection

Bruker X8 APEXII diffractometer
Absorption correction: multi-scan (*SADABS*; Sheldrick, 1996 ▶) *T*_min_ = 0.923, *T*_max_ = 0.961
10502 measured reflections
3699 independent reflections
3008 reflections with *I* > 2σ(*I*)
*R*_int_ = 0.033

#### Refinement

*R*[*F* ^2^ > 2σ(*F* ^2^)] = 0.042
*wR*(*F* ^2^) = 0.105
*S* = 0.99
3699 reflections
227 parameters
H-atom parameters constrained
Δρ_max_ = 0.20 e Å^−3^
Δρ_min_ = −0.19 e Å^−3^
Absolute structure: Flack (1983 ▶), 1483 Friedel pairs
Flack parameter: 0.00 (9)

### 

Data collection: *APEX2* (Bruker, 2008 ▶); cell refinement: *SAINT* (Bruker, 2008 ▶); data reduction: *SAINT*; program(s) used to solve structure: *SHELXS97* (Sheldrick, 2008 ▶); program(s) used to refine structure: *SHELXL97* (Sheldrick, 2008 ▶); molecular graphics: *X-SEED* (Barbour, 2001 ▶); software used to prepare material for publication: *publCIF* (Westrip, 2010 ▶).

## Supplementary Material

Crystal structure: contains datablocks global, I. DOI: 10.1107/S1600536810040328/xu5048sup1.cif

Structure factors: contains datablocks I. DOI: 10.1107/S1600536810040328/xu5048Isup2.hkl

Additional supplementary materials:  crystallographic information; 3D view; checkCIF report

## Figures and Tables

**Table 1:** Hydrogen-bond geometry (Å, °)

*D*—H⋯*A*	*D*—H	H⋯*A*	*D*⋯*A*	*D*—H⋯*A*
C14—H14⋯O1^i^	0.93	2.24	3.174 (3)	179

Symmetry code: (i) 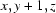.

**Fig. 1.**
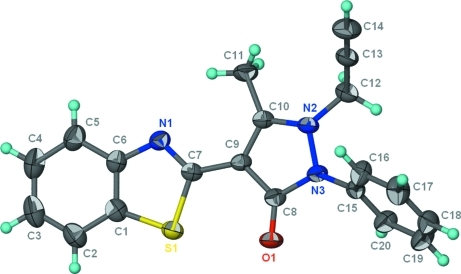
Thermal ellipsoid plot (Barbour, 2001) of C20H15N3OS at the 50% probability level; hydrogen atoms are drawn as arbitrary radius.

**Fig. 2.**
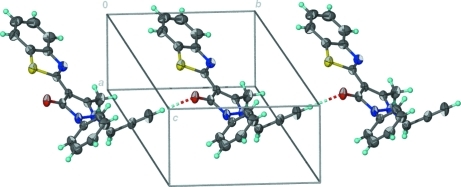
Hydrogen-bonded chain structure.

## supplementary crystallographic information

### Comment

(*E*)-4-(2,3-Dihydro-1,3-benzothiazol-2-ylidene)-3-methyl-1-phenyl-1*H*-pyrazol-5(4*H*)-one is an amine that can under a nucleophilic substitution with organo bromides to form 2-substituted derivatives if tetra-*n*-butyl ammonium bromide is used as catalyst. In this study, the compound is reacted with propargyl bromide to yield the title compound (Scheme I, Fig. 1).

### Experimental

To a solution of (*E*)-4-(2,3-dihydro-1,3-benzothiazol-2-ylidene)-3-methyl-1-phenyl-1*H*-pyrazol-5(4*H*)-one (1 g, 3.25 mmol) in DMF (50 ml), was added sodium carbonate (2.5 g, 23 mmol), tetra-*n*-butyl ammonium bromide (0.15 g, 1 mmol) and propargyl bromide (5.5 g, 46 mmol). The mixture was stirred for 24 h at room temperature. The solid material was removed by filtration and the solution was evaporated under reduced. The residue was washed with dichloromethane and hexane, and the recrystallized from ethanol to afford the title compound as yellow crystals.

### Refinement

Carbon-bound H-atoms were placed in calculated positions (C—H 0.93–0.97 Å) and were included in the refinement in the riding model approximation, with *U*(H) set to 1.2–1.5*U*_eq_(C).

### Crystal data

**Table:**

C_20_H_15_N_3_OS	*F*(000) = 720
*M**_r_* = 345.41	*D*_x_ = 1.347 Mg m^−^^3^
Orthorhombic, *P*2_1_2_1_2_1_	Mo *K*α radiation, λ = 0.71073 Å
Hall symbol: P 2ac 2ab	Cell parameters from 2503 reflections
*a* = 4.8221 (1) Å	θ = 2.4–22.9°
*b* = 9.3698 (2) Å	µ = 0.20 mm^−^^1^
*c* = 37.6990 (9) Å	*T* = 293 K
*V* = 1703.32 (6) Å^3^	Prism, yellow
*Z* = 4	0.40 × 0.20 × 0.20 mm

### Data collection

**Table:**

Bruker X8 APEXII diffractometer	3699 independent reflections
Radiation source: fine-focus sealed tube	3008 reflections with *I* > 2σ(*I*)
graphite	*R*_int_ = 0.033
φ and ω scans	θ_max_ = 27.1°, θ_min_ = 3.1°
Absorption correction: multi-scan (*SADABS*; Sheldrick, 1996)	*h* = −5→6
*T*_min_ = 0.923, *T*_max_ = 0.961	*k* = −11→11
10502 measured reflections	*l* = −47→46

### Refinement

**Table:**

Refinement on *F*^2^	Secondary atom site location: difference Fourier map
Least-squares matrix: full	Hydrogen site location: inferred from neighbouring sites
*R*[*F*^2^ > 2σ(*F*^2^)] = 0.042	H-atom parameters constrained
*wR*(*F*^2^) = 0.105	*w* = 1/[σ^2^(*F*_o_^2^) + (0.0613*P*)^2^] where *P* = (*F*_o_^2^ + 2*F*_c_^2^)/3
*S* = 0.99	(Δ/σ)_max_ = 0.001
3699 reflections	Δρ_max_ = 0.20 e Å^−^^3^
227 parameters	Δρ_min_ = −0.19 e Å^−^^3^
0 restraints	Absolute structure: Flack (1983), 1483 Friedel pairs
Primary atom site location: structure-invariant direct methods	Flack parameter: 0.00 (9)

### Fractional atomic coordinates and isotropic or equivalent isotropic displacement parameters (Å^2^)

**Table:**

	*x*	*y*	*z*	*U*_iso_*/*U*_eq_	
S1	0.55911 (13)	0.45298 (6)	0.105599 (16)	0.04655 (17)	
O1	0.9129 (4)	0.52382 (15)	0.16533 (4)	0.0487 (4)	
N1	0.5947 (4)	0.6723 (2)	0.06358 (5)	0.0445 (4)	
N2	1.1641 (4)	0.85264 (18)	0.14034 (5)	0.0422 (4)	
N3	1.1570 (4)	0.73580 (18)	0.16370 (5)	0.0408 (4)	
C1	0.3603 (5)	0.4536 (3)	0.06743 (6)	0.0469 (5)	
C2	0.1735 (6)	0.3514 (3)	0.05524 (7)	0.0599 (7)	
H2	0.1422	0.2680	0.0680	0.072*	
C3	0.0366 (6)	0.3767 (3)	0.02398 (8)	0.0681 (8)	
H3	−0.0894	0.3097	0.0155	0.082*	
C4	0.0833 (6)	0.4998 (3)	0.00504 (8)	0.0683 (8)	
H4	−0.0121	0.5142	−0.0161	0.082*	
C5	0.2662 (5)	0.6015 (3)	0.01643 (7)	0.0593 (7)	
H5	0.2961	0.6841	0.0033	0.071*	
C6	0.4073 (5)	0.5783 (3)	0.04845 (6)	0.0446 (5)	
C7	0.6919 (5)	0.6195 (2)	0.09303 (6)	0.0376 (5)	
C8	0.9769 (5)	0.6346 (2)	0.14978 (5)	0.0386 (5)	
C9	0.8913 (4)	0.6897 (2)	0.11590 (5)	0.0351 (4)	
C10	1.0182 (4)	0.8196 (2)	0.11109 (5)	0.0386 (5)	
C11	1.0201 (6)	0.9159 (3)	0.07987 (6)	0.0558 (7)	
H11A	0.9830	1.0119	0.0874	0.084*	
H11B	0.8800	0.8861	0.0634	0.084*	
H11C	1.1985	0.9120	0.0686	0.084*	
C12	1.3789 (5)	0.9610 (2)	0.14413 (7)	0.0479 (6)	
H12A	1.4965	0.9371	0.1641	0.057*	
H12B	1.4934	0.9620	0.1230	0.057*	
C13	1.2600 (5)	1.1024 (3)	0.14956 (6)	0.0494 (6)	
C14	1.1643 (7)	1.2142 (3)	0.15376 (8)	0.0657 (8)	
H14	1.0873	1.3042	0.1571	0.079*	
C15	1.2100 (5)	0.7561 (2)	0.20074 (5)	0.0383 (5)	
C16	1.0709 (5)	0.8595 (3)	0.21937 (7)	0.0534 (6)	
H16	0.9444	0.9189	0.2080	0.064*	
C17	1.1218 (6)	0.8741 (3)	0.25519 (7)	0.0625 (7)	
H17	1.0293	0.9440	0.2681	0.075*	
C18	1.3064 (6)	0.7867 (3)	0.27184 (7)	0.0588 (7)	
H18	1.3389	0.7973	0.2960	0.071*	
C19	1.4434 (6)	0.6842 (3)	0.25322 (7)	0.0629 (7)	
H19	1.5693	0.6248	0.2647	0.075*	
C20	1.3951 (5)	0.6682 (3)	0.21707 (6)	0.0505 (6)	
H20	1.4880	0.5983	0.2042	0.061*	

### Atomic displacement parameters (Å^2^)

**Table:**

	*U*^11^	*U*^22^	*U*^33^	*U*^12^	*U*^13^	*U*^23^
S1	0.0594 (3)	0.0375 (3)	0.0427 (3)	−0.0076 (3)	0.0002 (3)	0.0022 (2)
O1	0.0764 (10)	0.0299 (8)	0.0397 (9)	−0.0053 (8)	−0.0008 (9)	0.0053 (6)
N1	0.0509 (11)	0.0449 (11)	0.0376 (10)	−0.0009 (9)	0.0005 (9)	0.0036 (8)
N2	0.0573 (11)	0.0309 (9)	0.0386 (11)	−0.0056 (8)	0.0001 (9)	0.0042 (8)
N3	0.0598 (12)	0.0303 (9)	0.0323 (10)	−0.0002 (8)	−0.0029 (9)	0.0021 (8)
C1	0.0480 (12)	0.0497 (13)	0.0431 (13)	0.0012 (11)	0.0078 (10)	−0.0093 (11)
C2	0.0620 (15)	0.0589 (17)	0.0587 (17)	−0.0136 (13)	0.0045 (13)	−0.0111 (13)
C3	0.0521 (14)	0.086 (2)	0.0662 (19)	−0.0125 (15)	0.0019 (14)	−0.0292 (17)
C4	0.0551 (15)	0.096 (2)	0.0535 (17)	0.0027 (16)	−0.0097 (13)	−0.0122 (15)
C5	0.0586 (15)	0.0776 (19)	0.0418 (15)	−0.0006 (14)	−0.0034 (12)	0.0016 (13)
C6	0.0435 (12)	0.0544 (14)	0.0358 (12)	0.0030 (11)	0.0021 (10)	−0.0053 (10)
C7	0.0442 (11)	0.0346 (11)	0.0340 (12)	0.0014 (9)	0.0085 (9)	−0.0004 (9)
C8	0.0528 (13)	0.0297 (10)	0.0334 (12)	0.0042 (9)	0.0042 (9)	−0.0043 (8)
C9	0.0456 (11)	0.0301 (10)	0.0297 (10)	0.0026 (9)	0.0057 (9)	0.0008 (8)
C10	0.0488 (12)	0.0333 (10)	0.0336 (12)	0.0023 (8)	0.0052 (9)	0.0002 (9)
C11	0.0780 (17)	0.0472 (14)	0.0422 (14)	−0.0119 (12)	0.0012 (12)	0.0132 (10)
C12	0.0470 (12)	0.0391 (12)	0.0577 (15)	−0.0027 (10)	0.0044 (11)	0.0016 (11)
C13	0.0636 (15)	0.0378 (13)	0.0467 (15)	−0.0098 (12)	0.0002 (12)	0.0017 (10)
C14	0.093 (2)	0.0388 (14)	0.0652 (18)	0.0028 (14)	0.0063 (16)	0.0001 (12)
C15	0.0451 (12)	0.0360 (12)	0.0339 (12)	−0.0026 (9)	−0.0016 (9)	−0.0021 (9)
C16	0.0570 (13)	0.0545 (15)	0.0487 (14)	0.0157 (12)	−0.0105 (12)	−0.0128 (11)
C17	0.0676 (16)	0.0679 (18)	0.0519 (16)	0.0054 (14)	−0.0002 (14)	−0.0231 (13)
C18	0.0733 (17)	0.0678 (18)	0.0353 (14)	−0.0125 (15)	−0.0049 (13)	−0.0038 (12)
C19	0.0728 (17)	0.0678 (17)	0.0479 (15)	0.0121 (15)	−0.0133 (14)	0.0063 (13)
C20	0.0628 (15)	0.0459 (13)	0.0428 (13)	0.0117 (12)	−0.0012 (12)	−0.0030 (11)

### Geometric parameters (Å, °)

**Table:**

S1—C1	1.729 (2)	C9—C10	1.374 (3)
S1—C7	1.752 (2)	C10—C11	1.483 (3)
O1—C8	1.232 (2)	C11—H11A	0.9600
N1—C7	1.303 (3)	C11—H11B	0.9600
N1—C6	1.385 (3)	C11—H11C	0.9600
N2—C10	1.344 (3)	C12—C13	1.458 (3)
N2—N3	1.405 (2)	C12—H12A	0.9700
N2—C12	1.458 (3)	C12—H12B	0.9700
N3—C8	1.389 (3)	C13—C14	1.156 (4)
N3—C15	1.433 (3)	C14—H14	0.9300
C1—C6	1.389 (3)	C15—C20	1.362 (3)
C1—C2	1.393 (3)	C15—C16	1.372 (3)
C2—C3	1.371 (4)	C16—C17	1.379 (3)
C2—H2	0.9300	C16—H16	0.9300
C3—C4	1.375 (4)	C17—C18	1.362 (4)
C3—H3	0.9300	C17—H17	0.9300
C4—C5	1.368 (4)	C18—C19	1.361 (4)
C4—H4	0.9300	C18—H18	0.9300
C5—C6	1.403 (3)	C19—C20	1.391 (3)
C5—H5	0.9300	C19—H19	0.9300
C7—C9	1.449 (3)	C20—H20	0.9300
C8—C9	1.438 (3)		
			
C1—S1—C7	88.55 (12)	N2—C10—C9	109.20 (18)
C7—N1—C6	110.14 (19)	N2—C10—C11	120.51 (19)
C10—N2—N3	108.77 (17)	C9—C10—C11	130.3 (2)
C10—N2—C12	127.79 (18)	C10—C11—H11A	109.5
N3—N2—C12	119.92 (19)	C10—C11—H11B	109.5
C8—N3—N2	108.09 (17)	H11A—C11—H11B	109.5
C8—N3—C15	124.80 (18)	C10—C11—H11C	109.5
N2—N3—C15	120.19 (17)	H11A—C11—H11C	109.5
C6—C1—C2	120.9 (2)	H11B—C11—H11C	109.5
C6—C1—S1	109.94 (18)	C13—C12—N2	111.6 (2)
C2—C1—S1	129.1 (2)	C13—C12—H12A	109.3
C3—C2—C1	118.5 (3)	N2—C12—H12A	109.3
C3—C2—H2	120.8	C13—C12—H12B	109.3
C1—C2—H2	120.8	N2—C12—H12B	109.3
C2—C3—C4	120.8 (3)	H12A—C12—H12B	108.0
C2—C3—H3	119.6	C14—C13—C12	179.6 (3)
C4—C3—H3	119.6	C13—C14—H14	180.0
C5—C4—C3	121.9 (3)	C20—C15—C16	121.1 (2)
C5—C4—H4	119.1	C20—C15—N3	118.5 (2)
C3—C4—H4	119.1	C16—C15—N3	120.4 (2)
C4—C5—C6	118.3 (3)	C15—C16—C17	118.9 (2)
C4—C5—H5	120.8	C15—C16—H16	120.5
C6—C5—H5	120.8	C17—C16—H16	120.5
N1—C6—C1	115.4 (2)	C18—C17—C16	120.5 (3)
N1—C6—C5	124.9 (2)	C18—C17—H17	119.7
C1—C6—C5	119.7 (2)	C16—C17—H17	119.7
N1—C7—C9	125.0 (2)	C19—C18—C17	120.2 (2)
N1—C7—S1	115.93 (17)	C19—C18—H18	119.9
C9—C7—S1	119.08 (16)	C17—C18—H18	119.9
O1—C8—N3	123.5 (2)	C18—C19—C20	120.0 (3)
O1—C8—C9	130.8 (2)	C18—C19—H19	120.0
N3—C8—C9	105.66 (18)	C20—C19—H19	120.0
C10—C9—C8	107.89 (19)	C15—C20—C19	119.2 (2)
C10—C9—C7	128.25 (19)	C15—C20—H20	120.4
C8—C9—C7	123.76 (19)	C19—C20—H20	120.4
			
C10—N2—N3—C8	−6.4 (2)	O1—C8—C9—C7	2.1 (4)
C12—N2—N3—C8	−166.84 (19)	N3—C8—C9—C7	−177.20 (19)
C10—N2—N3—C15	−158.63 (19)	N1—C7—C9—C10	−1.5 (4)
C12—N2—N3—C15	41.0 (3)	S1—C7—C9—C10	−179.60 (17)
C7—S1—C1—C6	−0.38 (17)	N1—C7—C9—C8	174.4 (2)
C7—S1—C1—C2	−179.8 (2)	S1—C7—C9—C8	−3.7 (3)
C6—C1—C2—C3	0.0 (4)	N3—N2—C10—C9	6.0 (2)
S1—C1—C2—C3	179.4 (2)	C12—N2—C10—C9	164.5 (2)
C1—C2—C3—C4	0.2 (4)	N3—N2—C10—C11	−172.1 (2)
C2—C3—C4—C5	−0.1 (4)	C12—N2—C10—C11	−13.7 (3)
C3—C4—C5—C6	−0.3 (4)	C8—C9—C10—N2	−3.4 (2)
C7—N1—C6—C1	1.1 (3)	C7—C9—C10—N2	173.0 (2)
C7—N1—C6—C5	−179.4 (2)	C8—C9—C10—C11	174.5 (2)
C2—C1—C6—N1	179.1 (2)	C7—C9—C10—C11	−9.1 (4)
S1—C1—C6—N1	−0.3 (2)	C10—N2—C12—C13	81.7 (3)
C2—C1—C6—C5	−0.4 (3)	N3—N2—C12—C13	−122.0 (2)
S1—C1—C6—C5	−179.83 (18)	C8—N3—C15—C20	81.6 (3)
C4—C5—C6—N1	−178.9 (2)	N2—N3—C15—C20	−131.0 (2)
C4—C5—C6—C1	0.5 (4)	C8—N3—C15—C16	−96.6 (3)
C6—N1—C7—C9	−179.6 (2)	N2—N3—C15—C16	50.7 (3)
C6—N1—C7—S1	−1.4 (2)	C20—C15—C16—C17	0.2 (4)
C1—S1—C7—N1	1.08 (18)	N3—C15—C16—C17	178.4 (2)
C1—S1—C7—C9	179.36 (17)	C15—C16—C17—C18	−0.3 (4)
N2—N3—C8—O1	−175.2 (2)	C16—C17—C18—C19	0.1 (4)
C15—N3—C8—O1	−24.6 (3)	C17—C18—C19—C20	0.0 (4)
N2—N3—C8—C9	4.2 (2)	C16—C15—C20—C19	−0.1 (4)
C15—N3—C8—C9	154.8 (2)	N3—C15—C20—C19	−178.3 (2)
O1—C8—C9—C10	178.7 (2)	C18—C19—C20—C15	0.0 (4)
N3—C8—C9—C10	−0.6 (2)		

### Hydrogen-bond geometry (Å, °)

**Table:**

*D*—H···*A*	*D*—H	H···*A*	*D*···*A*	*D*—H···*A*
C14—H14···O1^i^	0.93	2.24	3.174 (3)	179

Symmetry codes: (i) *x*, *y*+1, *z*.
